# Dynamic feedback bit-level image privacy protection based on chaos and information hiding

**DOI:** 10.1038/s41598-024-53325-4

**Published:** 2024-03-08

**Authors:** Jinlong Zhang, Heping Wen

**Affiliations:** 1 School of information technology and management, Hunan University of Finance and Economics, Changsha, 410205 China; 2 University of Electronic Science and Technology of China, Zhongshan Institute, Zhongshan, 528402 China; 3 University of Electronic Science and Technology of China, Chengdu, 611731 China

**Keywords:** Energy science and technology, Engineering, Physics

## Abstract

Bit is the most basic unit of a digital image in the spatial domain, and bit-level encryption is regarded as an important technical means for digital image privacy protection. To address the vulnerability of image privacy protection to cryptographic attacks, in this paper, a bit-level image privacy protection scheme using Zigzag and chain-diffusion is proposed. The scheme uses a combination of Zigzag interleaving scrambling with chaotic sequences and chain-diffusion method images are encrypted at each bit level, while using non-sequential encryption to achieve efficient and secure encryption. To balance security and efficiency, the encryption strategy for each bit layer is weighted. The chaos-based sequences used for encryption depend on the previous hash value, thus the effect of chain-diffusion is achieved. To further enhance the encryption effect, a non-sequential encryption technique by non-linearly rearranging the bit cipher image is employed, so that the attacker cannot crack the protection scheme by analyzing the encrypted image. The ciphertext image hidden by discrete wavelet transform (DWT) also provides efficient encryption, higher level of security and robustness to attacks. This technology provides indistinguishable secret data embedding, making it difficult for attackers to detect or extract hidden information. Experimental results show that this scheme can effectively protect the confidentiality of the image and can resist various common cryptographic attacks. The scheme proposed in this paper is a preferred digital image privacy protection technology, so it has broad application prospects in image secure transmission occasions.

## Introduction

Under the vigorous development of computer communication and network technology, diversified data and information can continuously, widely and rapidly flow through the network, thus giving rise to new needs in the field of data transmission, especially the concern for the transmission security environment^1–5^. In an intuitive and common form, images carry a large amount of sensitive information as the carrier of information transmission^6–10^. Therefore, image encryption technology^11–16^ can efficiently secure key information and prevent information leakage during data transmission. A variety of encryption methods have been proposed, including quantum cipher^17–19^, thumbnail-preserving encryption^20–23^, biological coding^24–26^, discrete wavelet transform^27–29^, Fourier transform^30–32^, discrete cosine transform^33–35^, bit-level encryption^36–38^, chaos theory^39–44^ and so on^45–54^. Especially, chaos algorithm is widely used and highly^55–60^ respected in the field of image encryption due to its unpredictability, pseudo-randomness, and high sensitivity to the initial value^61–66^.

Throughout the international situation, many scholars have achieved a series of important theoretical and practical achievements in using chaos for image encryption^67–74^. In 2021, Ref.^75^ proposed a new parallel processing method for chaos-based image encryption. The scheme splits the image data and creates encrypted threads that process the partitions in parallel using the same chaotic cipher. Based on the additional chaotic function and the XOR, shift operation, which results in encryption. Test results show that the proposed architecture is faster than the base cipher and other advanced algorithms and passes the security test with good robustness. In 2022, Ref.^76^ proposed a new method for RGB color image encryption based on chaotic cross-channel pixel and bit scrambling. By utilizing the chaos principle, along with the cross-channel pixel and bit scrambling parameters, as well as the required parameters during the diffusion phase, the objective of image encryption can be achieved. Experimental findings demonstrate that this encryption algorithm effectively withstands different common cryptographic attacks and possesses robust anti-interference capabilities, thus reducing paper duplication. In 2023, Ref.^77^ proposed a dynamic RNA-encoded color image encryption scheme based on a chain feedback structure. The color image is encrypted using a chaotic sequence based on plaintext correlation for each color component and the color-coded image is obtained through RNA dynamic encoding and other operations. The results of the experiment demonstrate that the encryption algorithm exhibits outstanding encryption effectiveness and security performance in the face of different attacks. From a security perspective, existing bit-level chaotic encryption algorithms need further improvement, mainly because (1) the present algorithms are susceptible to chosen-plaintext attacks or chosen-ciphertext attacks as the key employed for generating chaotic sequences lacks correlation with the original image; (2) the granularity of encryption units of existing algorithms is coarse; (3) existing bit planes decomposition algorithms do not consider the correlation between each sliced plane after decomposition. Within the current realm of research on chaotic image encryption, the performance of chaos and algorithms significantly influences the security and efficiency of cryptographic systems. It is imperative and pressing to investigate a novel image encryption algorithm that relies on chaotic mapping construction to withstand various illicit attacks.

In this research paper, we present a Zigzag and chain-diffusion scheme for Bit-level image privacy protection. Our experimental findings demonstrate the algorithm’s outstanding performance in encryption, with good efficiency and the ability to withstand various unauthorized attacks on image encryption. The main innovations and contributions of this paper are as follows:

(A) The image encryption method using Discrete Wavelet Transform (DWT) involves efficient encryption, heightened security, and resistance against attacks. This technique makes embedded secret data indistinguishable, challenging for attackers to detect or extract. Additionally, DWT-based hiding ensures the integrity of hidden data even under common attacks like noise addition or compression. In conclusion, DWT hiding is an effective approach for securely protecting sensitive information in images while preserving their visual quality.
(B) The existing image encryption algorithms are not structured rationally enough, which leads to their insufficient security against plaintext-type attacks. For this reason, this color image encryption algorithm proposes a plaintext and intermediate ciphertext association mechanism and also adopts chain diffusion to effectively enhance the resistance to cryptographic attacks.
(C) Pixel-level image encryption is so coarse in granularity that it is not secure enough, and traditional bit-level encryption is too complex to meet the efficiency requirements. To cope with these challenges, this paper proposes a new strategy. We adopt an elastic processing unit in the weighted bit plane, which effectively balances the tension between security and efficiency.
(D) Many of the existing encryption methods rely on pixel-level encryption, which leads to relatively weak encryption granularity, and pixel-level scrambling poses certain security risks. For the encryption of color images, we adopt a bit-level encryption strategy and further enhance the security of the encryption algorithm using forward-and-backward XOR and Zigzag interleaving scrambling. The experimental results show that this algorithm has a significant security improvement.

The rest of the paper is organized as follows: “Relevant theories” briefly describes the bit plane decomposition of chaotic systems as well as non-sequential encryption algorithms. “The proposed encryption algorithm” presents the encryption algorithm designed in this paper. “Analysis and discussion of experimental results” gives experimental and simulation results. The last section concludes the paper.

##  Relevant theories

### HLSE chaotic system

This paper uses a HLSE chaotic system^78^. The specific equation is expressed as follows:

1 $$\begin{aligned} x(n)=\gamma sin(\pi \cdot e^{x(n-1)})[1-sin(\pi \cdot e^{x(n-1)})]mod 1 \end{aligned}$$

where *mod* denotes the modulo operation, $\gamma$ denotes the control parameter, whose range is (0,$\infty$), *x*(0) denotes the initial value, *x*(*n*) denotes the generated chaotic sequence, whose range is (0,1), at the same time, the system will have chaotic characteristics when $\gamma$>3.

### Discrete wavelet transform

The discrete wavelet transform (DWT) is a powerful mathematical tool used in signal processing and image compression. It decomposes a signal or an image into different frequency components, allowing for both time and frequency domain analysis. DWT is widely applied in various fields, including image processing, data compression, and denoising.

**Figure 1:**
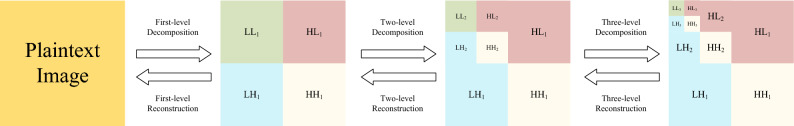
Flow chart of DWT algorithm.

The wavelet transform operates by iteratively refining the signal across multiple scales, achieving this through a series of scaling and translation operations. This progressive refinement process culminates in a remarkable outcome: a high-frequency time division and a low-frequency frequency division of the signal. This unique characteristic enables the wavelet transform to automatically adapt to the intricate demands of time-frequency signal analysis. The schematic diagram of the image wavelet decomposition is shown in Fig. 1. The (DWT) can be represented by the following formula:

2 $$\begin{aligned} W(a,b)=\sum \limits _{n=0}^{N-1}{x(n)\cdot {{\psi }_{a,b}}(n)} \end{aligned}$$

where *W*(*a*, *b*) represents the transformed coefficient, with *a* and *b* denoting the scale and translation parameters, respectively. These parameters are utilized to control the shape and position of the wavelet function. *x*(*n*) corresponds to the discrete sample values of the input signal. $\psi _{a,b}(n)$ represents the wavelet function, which is dependent on the scale parameter *a* and translation parameter *b*.

### Bit plane decomposition

**Figure 2:**
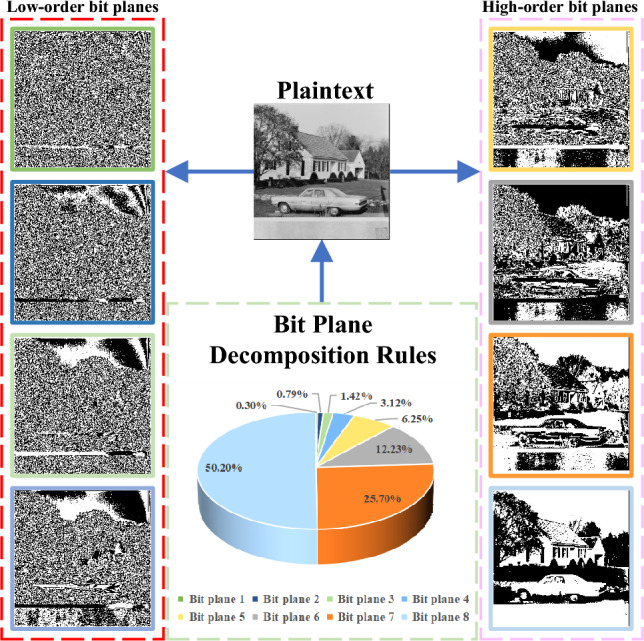
Schematic diagram of Bit decomposition.

A digital image is an image obtained by digitizing an analog image with pixels as its basic elements, which can be stored and processed by a digital computer or digital circuit. A bit is a unit of information and the smallest unit of measurement of bits and information in a binary number. Bit plane decomposition is the process of converting the pixel values of a digital image into binary, which in turn can be divided into 8-bit planes. Taking the digital image *P* as an example, the bit plane decomposition can be expressed as:

3 $$\begin{aligned} P=\sum _{k=1}^{8} 2^{k-1} P_k=P_1+2P_2+2^{2}P_3+2^{3}P_4+2^{4}P_5+2^{5}P_6+2^{6}P_7+2^{7}P_8 \end{aligned}$$

where *k*=[1,2,3,...,7,8], $P(i,j)\in$ $\mathbb {Z}_{256}$, $P_k(i,j)\in$ $Z_{2}$. $P_k$ denotes the *k*-th bit plane, $P_8$ denotes the highest bit plane and $P_1$ denotes the lowest bit plane. Taking the “house” grayscale map as an example, the bit plane decomposition diagram is shown in Fig. 2.

## The proposed encryption algorithm

**Figure 3:**
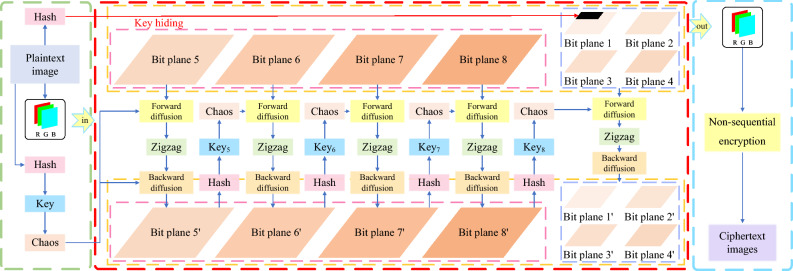
Flow chart of encryption algorithm.

To solve the existing problems, this paper proposes an image encryption scheme based on chain encryption with image bit-level layering, thus improving the efficiency and security of the encryption algorithm and at the same time has a certain ability to resist cryptographic attacks. The details of the specific encryption algorithm are shown in the following Fig. 3.

### Chaotic initial value confusion and sequence preprocessing

In this section, the association between plaintext and ciphertext is realized using the hash MD5 function, which can effectively improve the algorithm’s ability to resist the chosen plaintext attack and the chosen ciphertext attack because of its unidirectional and collision-proof properties. Meanwhile, in cryptography, the original chaotic sequence initially generated cannot be directly used as an encryption tool and needs to be manipulated by mathematical methods to preserve its chaotic properties and make each of its values fall within the interval required by the algorithm. Finally, the two chaotic sequences obtained by processing are represented by $S_1$ and $S_2$.

### Chain diffusion function

The specific operational details of each plane in this paper are defined as a custom function $C=Chain\_encrypt(I,key)$, where *I* denotes the input plaintext image, *C* denotes the output ciphertext image, and *key* denotes the initial key of the chaotic sequence required to encrypt the next image. The function consists of three parts: 2D forward XOR diffusion, Zigzag interleaving scrambling, and 2D backward XOR diffusion. The specific operations are shown below:

Step 1: 2D forward XOR diffusion.

The generated chaotic sequence $S_1$ is reconstituted into a chaotic matrix of size $H\times W$, where $m=[1,2,3,\dots ,H], n=[1,2,3,\dots ,W]$. The specific input image *I* is encrypted as follows:

4 $$\begin{aligned} {\left\{ \begin{array}{ll} C_1(1,1)=I(1,1)\oplus X(1,1)\\ C_1(1,j)=I(1,j)\oplus X(1,j)\oplus C_1(1,j-1)\\ C_1(i,1)=I(i,1)\oplus X(i,1)\oplus C_1(i-1,1)\\ C_1(i,j)=I(i,j)\oplus X(i,j)\oplus C_1(i-1,j-1)\\ \end{array}\right. } \end{aligned}$$

where $i=[2,\dots ,H],j=[2,\dots ,W]$. After the above 2D forward XOR diffusion, a preliminary encrypted image $C_1$ is obtained.

Step 2: Zigzag interleaved scrambling.

The initial encrypted image $C_1$ is disrupted by Zigzag interleaved scrambling to get the image $C_2$. For the scanning process, the first element in the upper left corner of the original encrypted image $C_1$ of size $H\times W$ is selected as the starting point. Then, the first scanning is performed until the $\frac{H\times W}{2}$-th element is scanned and each scanned element is integrated into array $V_1$. Similarly, the lower half is scanned starting from the first element in the lower right corner, and each scanned element is integrated into array $V_2$. and it will be reconstructed into a new matrix $C_2$, which size of $H\times W$, in an interleaved.

Step 3: 2D backward XOR diffusion.

It can be seen from step 1 that the forward XOR diffusion starts from the upper left corner to the lower right corner. Similarly, the backward XOR diffusion starts from the lower right corner to the upper left corner. After the above 2D backward XOR diffusion, the encrypted image *P* is obtained.

### The proposed image privacy protection algorithm

#### Encryption algorithm section

**Figure 4:**
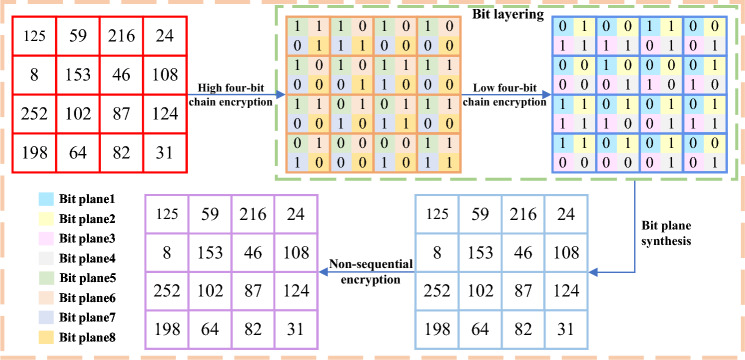
Flow chart of specific steps for encryption.

This section proposes a multi-bit hierarchical and chained encryption image encryption scheme based on image features. Taking the encrypted image with size of $H\times W$ as an example, the schematic diagram and encryption steps of the algorithm are shown in Fig. 4.

Step 1: Decompose bit plane.

After input the image *Q* and splitting it according to the three channels R, G, B, three grayscale images $Q_R$, $Q_G$ and $Q_B$ are obtained, which are respectively subjected to bit plane layering with the following equations:

5 $$\begin{aligned} {\left\{ \begin{array}{ll} Q_{Rk}=bitget(Q_R,k)\\ Q_{Gk}=bitget(Q_G,k)\\ Q_{Bk}=bitget(Q_B,k)\\ \end{array}\right. } \end{aligned}$$

where the *bitget*(*P*, *k*) function denotes the return of the bit value of the *k*-th layer in *P*, $$Q_{Rk}$$, $$Q_{Gk}$$ $$Q_{Bk}$$ denotes the image obtained after layering $$Q_R$$, $$Q_G$$, $$Q_B$$, and *k* denotes the *k*-th bit plane, $$k=[1,2,3,\dots ,7,8]$$. On this basis, the R-channel is used as an example to generate eight layered images $$Q_{R1}$$, $$Q_{R2}$$, $$Q_{R3}$$, $$Q_{R4}$$, $$Q_{R5}$$, $$Q_{R6}$$, $$ Q_{R7}$$, $$Q_{R8}$$ and the G-channel and B-channel operations are the same as the R-channel.

Step 2: Hide the original image feature values.

To facilitate the decryption operation by the receiver, the hash value of the original image needs to be stored in the first row of the layered image $Q_{R1}$. It is worth noting that, as shown in the analysis in “Bit plane decomposition”, the first layer after bit plane layering contains very little information. Taking an image of size $256 \times 256$ as an example, the proportion of feature values in the original image is only 0.000586%. Even if the decrypted image is enlarged, it is difficult to observe the difference with the naked eye.

Step 3: Encrypt layer 5.

The encrypted ciphertext image $C_{R5}$ is reprocessed to obtain $key_5$, which is used for the next round of encryption. The formula is as follows:

6 $$\begin{aligned} C_{R5}=Chain\_encrypt(Q_{R5},key_1) \end{aligned}$$

Step 4: Encrypt layers 6–8.

Images $Q_{R6}$, $Q_{R7}$, $Q_{R8}$ are processed in the same way as Step 3, with the formulas shown below:

7 $$\begin{aligned} {\left\{ \begin{array}{ll} C_{R6}=Chain\_encrypt(Q_{R6},key_5)\\ C_{R7}=Chain\_encrypt(Q_{R7},key_6)\\ C_{R8}=Chain\_encrypt(Q_{R8},key_7)\\ \end{array}\right. } \end{aligned}$$

Step 5: Encrypt layers 1–4.

For the low-order bit plane, which contains only a small amount of image information, the same key sequence will be used to perform the encryption operation on these four layers. Similarly, the $C_{R8}$ eigenvalue is obtained and processed to obtain $key_8$. The encryption of these four layers can be expressed as:

8 $$\begin{aligned} {\left\{ \begin{array}{ll} C_{R1}=Chain\_encrypt(Q_{R1},key_8)\\ C_{R2}=Chain\_encrypt(Q_{R2},key_8)\\ C_{R3}=Chain\_encrypt(Q_{R3},key_8)\\ C_{R4}=Chain\_encrypt(Q_{R4},key_8)\\ \end{array}\right. } \end{aligned}$$

Step 6: Composite bit plane.

The encrypted image $$C_{R1}$$, $$C_{R2}$$, $$C_{R3}$$, $$C_{R4}$$, $$C_{R5}$$, $$C_{R6}$$, $$ C_{R7}$$, $$C_{R8}$$ is subjected to layers synthesis with the following formula:

9 $$\begin{aligned} C_R=\sum _{i=1}^{8} 2^{i-1} C_{Ri}=2^{0} C_{R1}+2^{1} C_{R2}+2^{2}C_{R3}+2^{3}C_{R4}+2^{4}C_{R5}+2^{5}C_{R6}+2^{6}C_{R7}+2^{7}C_{R8} \end{aligned}$$

where $C_{R}$ denotes the final ciphertext image of the R-channel after reduction.

Similarly, the ciphertext image $C_{G}$, $C_{B}$ can be obtained after chain encryption of the G-channel and B-channel, the ciphertext image *C* can be obtained after three-channel reduction.

Step 7: Non-sequential encryption.

In order to achieve the diffusion characteristics of encryption algorithms, most image encryption algorithms adopt the method of changing the current pixel from the previous pixel. However, using fixed order pixel processing may reduce encryption performance and provide attackers with a large amount of information. To solve this problem, we adopted a non sequential encryption algorithm that uses random and secret access mechanisms to process pixels. The processing order is determined by the generated chaotic sequence. As a result, each pixel may be influenced not only by pixels within the same color plane but also by pixels from different color planes. The encryption and decryption operations are as follows:

10 $$\begin{aligned} C_{i,j,k}= & {} {\left\{ \begin{array}{ll} (S_{i,j,k}+C_{M,N,3}+A_{i,j,k})mod F \quad &{}if\quad i=1,j=1,k=1,\\ (S_{i,j,k}+C_{M,N,k-1}+A_{i,j,k})mod F \quad &{}if\quad i=1,j=1,k\ne 1,\\ (S_{i,j,k}+C_{M,j-1,k}+A_{i,j,k})mod F \quad &{}if\quad i=1,j\ne 1,\\ (S_{i,j,k}+C_{i-1,N,k}+A_{i,j,k})mod F \quad &{}if\quad i\ne 1,\\ \end{array}\right. } \end{aligned}$$

11 $$\begin{aligned} S_{i,j,k}= & {} {\left\{ \begin{array}{ll} (C_{i,j,k}-C_{M,N,3}-A_{i,j,k})mod F \quad &{}if\quad i=1,j=1,k=1,\\ (C_{i,j,k}-C_{M,N,k-1}-A_{i,j,k})mod F \quad &{}if\quad i=1,j=1,k\ne 1,\\ (C_{i,j,k}-C_{M,j-1,k}-A_{i,j,k})mod F \quad &{}if\quad i=1,j\ne 1,\\ (C_{i,j,k}-C_{i-1,N,k}-A_{i,j,k})mod F \quad &{}if\quad i\ne 1,\\ \end{array}\right. } \end{aligned}$$

where *mod* denotes the modulo operation, *P* is the input color image, *A* is the chaos matrix generated from the chaotic sequence and *F* denotes the number of pixel values in each color image *P*. The encryption step has been completely completed and the final ciphertext has been obtained.

#### Embedding a mask image

**Figure 5:**
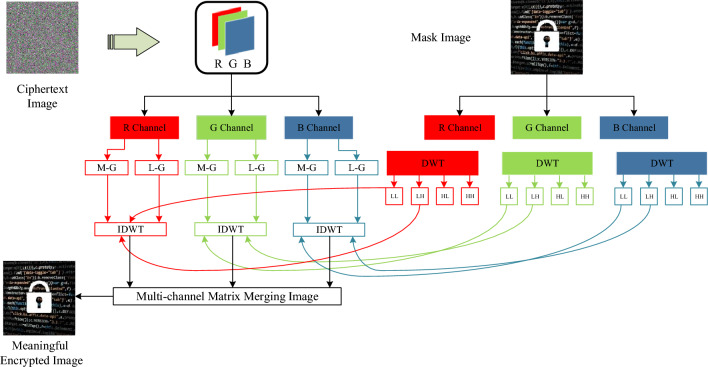
Schematic diagram of the ciphertext image embedding process.

To convert a random ciphertext image into a meaningful output image, a DWT is used in the proposed of the proposed encryption scheme. The masking of the new image onto the ciphertext image according to the following steps:

1. Take a mask image having meaningful information of size $2M \times 2N \times 3$.
2. Apply DWT to each color component of a mask image and extract four frequency sub-bands.
3. Now, split each pixel value of the pre-ciphertext image into its groups: (a) *LSB*-group and (b) *MSB*-group. For example, a pixel value having a grayscale value equal to 152 ($Gra{{y}_{dec}}$ = 152), its binary version will be $Gra{{y}_{bin}}$ = 10011000. The *LSB* and *MSB* group of the binary value will be ${{G}_{1}}$ = 1001 and ${{G}_{2}}$ = 1000, respectively.
4. Similarly, step 3 will be repeated for each pixel until it reaches position (*M*, *N*) for each color component. The *LSB*-group $(L - G)$ and *MSB*-group $(M - G)$ matrices are given in Eqs. (9) and (10), respectively.
  12 $$\begin{aligned} L-G= & {} \left[ \begin{aligned}&\begin{matrix} {{(01010000)}_{1,1}} &{} \ldots &{} {{\text {(11100000)}}_{1,N}} \\ {{(11000000)}_{2,1}} &{} \ldots &{} {{\text {(10100000)}}_{2,N}} \\ \vdots &{} \ddots &{} \vdots \\ {{(11000000)}_{M-1,1}} &{} \cdots &{} {{(10110000)}_{M-1,N-1}} \\ {{(10110000)}_{M,1}} &{} \cdots &{} {{(11100000)}_{M,N}} \\ \end{matrix} \\ \end{aligned} \right] \end{aligned}$$
  13 $$\begin{aligned} {{M}_{G}}= & {} \left[ \begin{aligned}&\begin{matrix} {{(00001110)}_{1,1}} &{} \ldots &{} {{\text {(00001000)}}_{1,N}} \\ {{(00001100)}_{2,1}} &{} \ldots &{} {{\text {(00001000)}}_{2,N}} \\ \vdots &{} \ddots &{} \vdots \\ {{(00001110)}_{M-1,1}} &{} \cdots &{} {{(00001111)}_{M-1,N-1}} \\ {{(00001111)}_{M,1}} &{} \cdots &{} {{(00001000)}_{M,N}} \\ \end{matrix} \\ \end{aligned} \right] \end{aligned}$$
5. The extracted high-frequency sub-bands (HL and HH) will be replaced with the two binary groups $(L-GandM-G)$.
6. After replacing the sub-bands, take the inverse DWT(IDWT) to restore the original mask image (${{I}_{{{R}_{mask}}}}$). This ${{I}_{{{R}_{mask}}}}$ image will be transmitted as a meaningful encrypted image. The block diagram of the proposed embedding process is displayed in Fig. 5.

#### Decryption algorithm section

**Figure 6:**
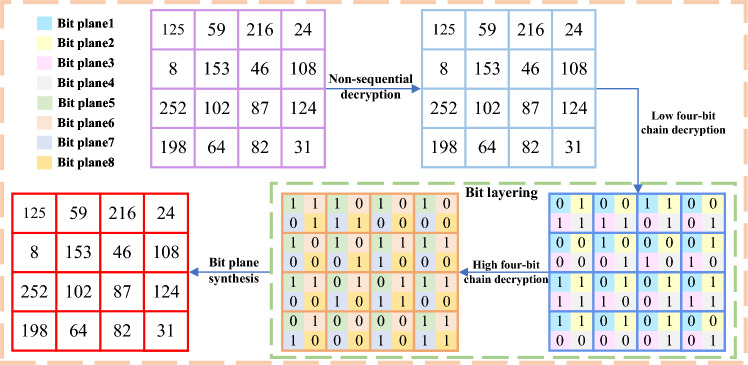
Flow chart of specific steps for decryption.

Decryption can be regarded as the inverse process of encryption, where the final ciphertext image is first decrypted in a non-sequential manner and then the ciphertext image *C* is divided into *R*, *G*, *B* channels to obtain $C_{R}$, $C_{G}$, $C_{B}$. The specific operation steps are shown in the following Fig. 6.

Take the R-channel as an example, perform bit plane layering to obtain 8-bit planes $$C_{R1}$$, $$C_{R2}$$, $$C_{R3}$$, $$C_{R4}$$, $$C_{R5}$$, $$C_{R6}$$, $$ C_{R7}$$, $$C_{R8}$$, the G-channel and B-channel operate the same. Extract the original image feature values stored in the first row of the bit-plane layering and process them to obtain $$key_1$$. The encrypted image of the fifth plane can be decrypted and the image is obtained $$Q_{R5}$$. The obtained image is continued to be used for decryption until eight plaintext images after bit-plane layering are obtained, and finally, the initial plaintext image *P* is obtained.

##  Analysis and discussion of experimental results

### Experimental environment

The proposed algorithm was validated on a PC host computer equipped with MATLAB R2023a experimental software. The PC is equipped with an 11th Gen Intel Core i7-11800H CPU operating at 2.30 GHz. The PC has 32 GB of RAM. The image data selected for the experiments are from the standardized test image database USC-SIPI.

### Statistical analysis

#### Histogram analysis

**Figure 7:**
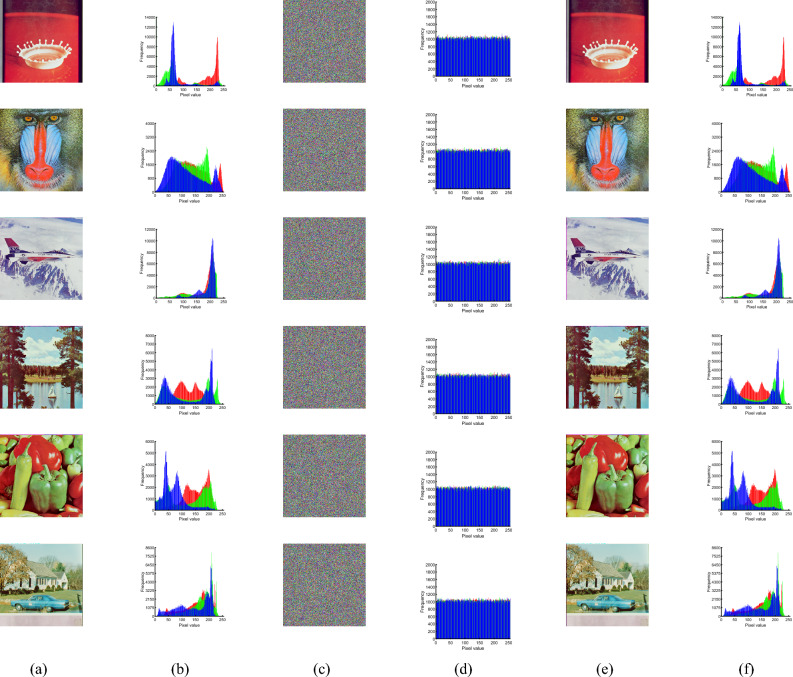
Histogram comparison: (**a**) plaintext image, (**b**) plaintext image histogram, (**c**) ciphertext image, (**d**) ciphertext image histogram, (**e**) decrypted image, (**f**) decrypted image histogram.

Histograms display statistical information about an image, visualizing the distribution of individual values in the image. The histograms of plaintext images exhibit distinct statistical patterns, and attack schemes that target statistical patterns are known as statistical analysis attacks. We compute and plot the histograms of the original image and the ciphertext. Figure 7a depicts the selection of six plaintext images with various sizes, which are then encrypted to generate the corresponding ciphertext images illustrated in Fig. 7c. The histograms of the images before and after encryption and decryption are presented in Fig. 7b,f, respectively. The histograms of the plaintext images show certain statistical regularity, while the encrypted images show a noise-like distribution, and the statistical properties of the histograms show a uniform distribution. This well hides the key information of the image, thus demonstrating the ability of the proposed algorithm to resist statistical analysis attacks.

#### Adjacent pixel correlation analysis

Usually, plaintext images have pixels with high neighborhood correlation and exhibit a statistical property. And a good encryption algorithm should make the encrypted image achieve de-correlation between its neighboring pixels.

**Figure 8:**
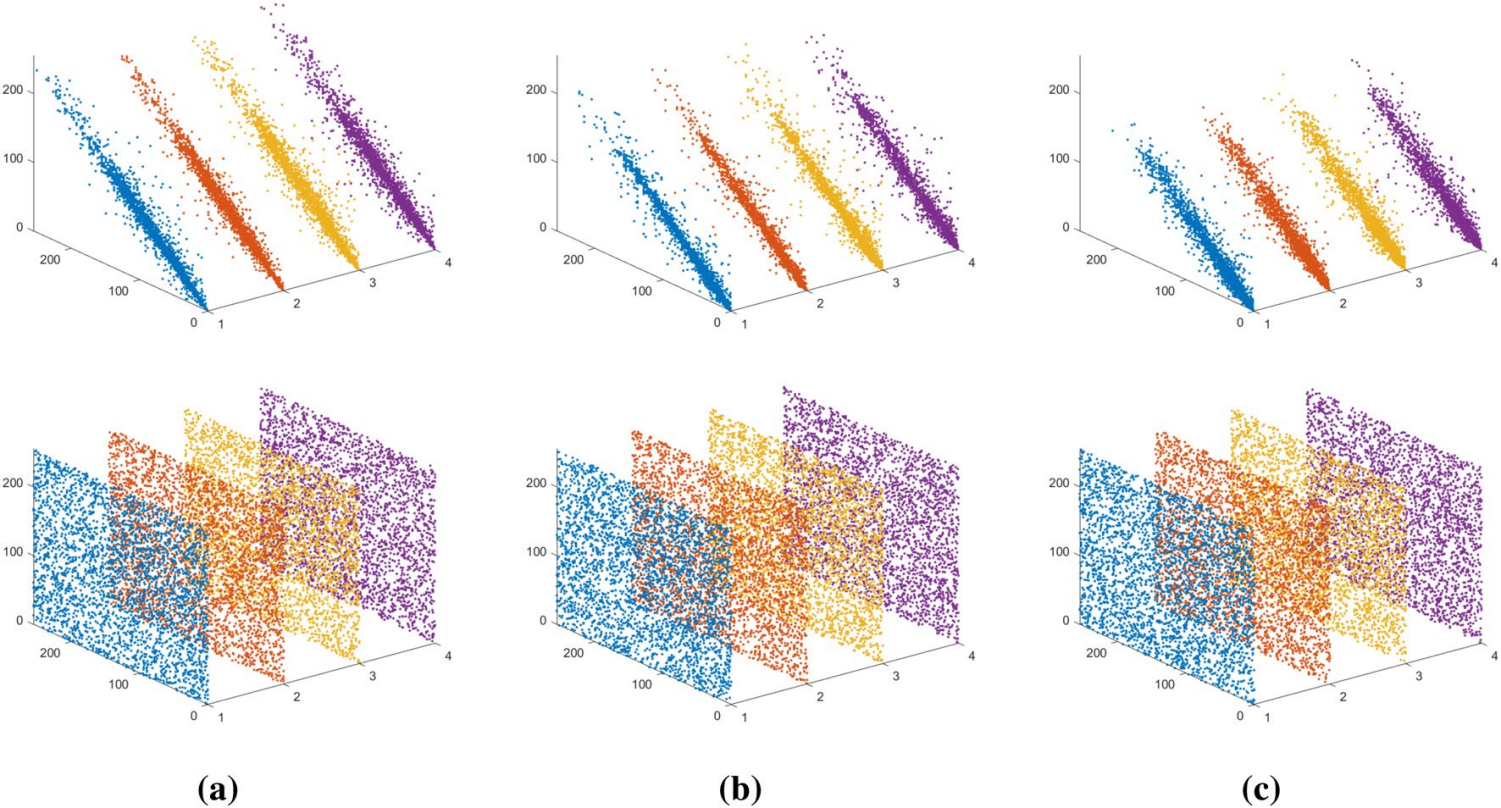
Adjacent pixel correlation analysis: (**a**) R channel, (**b**) G channel, (**c**) B channel.

We calculated and compared the correlation between adjacent pixels in both the plaintext and ciphertext images. This was achieved through the following steps. Initially, 3000 pairs were randomly selected of adjacent pixel coordinates from both the plaintext and ciphertext images. Subsequently, we calculate the correlation coefficients for adjacent pixels in the horizontal, vertical, and diagonal, as well as the antidiagonal directions. The correlation coefficients are calculated using the following formula:

14 $$\begin{aligned} r_{xy}=\frac{\sum _{i=1}^{M} (x_{i}-{\frac{1}{M}\sum _{j=1}^{M}}{x_{j}})(y_{i}-{\frac{1}{M}\sum _{j=1}^{M}}{y_{j}})}{{\sqrt{\sum _{i=1}^{M} (x_{i}-{\frac{1}{M} \sum _{j=1}^{M}}{x_{j}})^{2}}}{\sqrt{\sum _{i=1}^{M} (y_{i}-{\frac{1}{M}\sum _{j=1}^{M}}{y_{j}})^{2}}}} \end{aligned}$$

where $x_i$ and $y_i$ constitute the *i* pair of horizontal/vertical/diagonal/antidiagonal neighboring pixels and *M* is the total number of horizontal/vertical/diagonal/antidiagonal neighboring pixels. The adjacent pixel correlation data of the encrypted image is shown in Fig. 8 and Table 1. From the experimental data, the correlation coefficient of the plaintext image is close to 1, while the correlation coefficient of the ciphertext image is approximately equal to 0. This indicates that the proposed encryption scheme generates images with de-correlated neighboring pixels. Therefore, the proposed scheme in this paper is secure against statistical attacks.

**Table 1:** Comparison results of correlation coefficients of adjacent pixels.

Component	Direction	Plaintext image	Proposed
R	Horizontal	0.9591	0.0017
	Vertical	0.9757	− 0.0157
	Diagonal	0.9495	0.0176
	Anti-diagonal	0.9595	− 0.0027
G	Horizontal	0.9584	− 0.0080
	Vertical	0.9759	− 0.0093
	Diagonal	0.9453	− 0.0153
	Anti-diagonal	0.9466	0.0041
B	Horizontal	0.9572	− 0.0074
	Vertical	0.9605	0.0279
	Diagonal	0.9387	0.0054
	Anti-diagonal	0.9353	0.0115

#### Differential statistical analysis

The number-of-pixels change rate (NPCR) and uniform average change intensity (UACI) are frequently employed to assess the resilience of cryptosystems against performance differential attacks. Typically, an attacker modifies the original image by introducing small alterations and subsequently encrypts both the original image and the modified version using the proposed algorithm. This allows the attacker to examine the correlation between the plaintext image and the ciphertext image through a differential attack. To evaluate the resistance of the proposed algorithm against differential attacks, we calculated and compared the NPCR and UACI values using the following formulas:

15 $$\begin{aligned} {\left\{ \begin{array}{ll} NPCR = \frac{1}{H} \times \frac{1}{W} \times \sum \limits _{i=1}^{H}\sum \limits _{j=1}^{W}D(i,j)\times 100\%\\ UACI = \frac{1}{H} \times \frac{1}{W} \times \sum \limits _{i=1}^{H} \sum \limits _{j=1}^{W} \frac{|v_{1}(i,j) - v_{2}(i,j)|}{255} \times 100\%\\ \end{array}\right. } \end{aligned}$$

where $H\times W$ is the size of the image, $v_1$ and $v_2$ are the ciphertext image before and after the plaintext image is changed by one pixel respectively. *D* can be defined by the following equation:

16 $$\begin{aligned} D={\left\{ \begin{array}{ll} 0 &{} \quad if\quad v_{1}(i,j) = v_{2}(i,j)\\ 1 &{} \quad if\quad v_{1}(i,j) \ne v_{2}(i,j)\\ \end{array}\right. } \end{aligned}$$

The results of the algorithm, calculated using Eq. (15), are presented in Table 2. The analysis of Table 2 reveals that NPCR and UACI closely approximate their ideal values of 99.6% and 33.4%, respectively.

**Table 2:** NPCR and UACI values.

Images	Description	Size	NPCR ($\%$)	UACI ($\%$)
4.1.01	Female (NTSC test image)	256 $\times$ 256	99.6078	33.4188
4.1.04	Female	256 $\times$ 256	99.6048	33.4412
4.1.05	House	256 $\times$ 256	99.5956	33.3870
4.1.06	Tree	256 $\times$ 256	99.6048	33.4535
4.1.07	Jelly beans	256 $\times$ 256	99.5987	33.4508
4.2.01	Splash	512 $\times$ 512	99.6010	33.4293
4.2.03	Mandrill (a.k.a. Baboon)	512 $\times$ 512	99.6037	33.4362
4.2.05	Airplane (F-16)	512 $\times$ 512	99.6029	33.4287
4.2.06	Sailboat on lake	512 $\times$ 512	99.6056	33.4204
4.2.07	Peppers	512 $\times$ 512	99.6037	33.4246
5.1.09	Moon surface	256 $\times$ 256	99.6155	33.3541
5.1.10	Aerial	256 $\times$ 256	99.6155	33.4622
5.1.11	Airplane	256 $\times$ 256	99.6094	33.4382
5.1.12	Clock	256 $\times$ 256	99.6002	33.3685
5.1.14	Chemical plant	256 $\times$ 256	99.6063	33.5118
5.3.01	Male	512 $\times$ 512	99.6022	33.4692
7.1.01	Truck	512 $\times$ 512	99.6181	33.3533
7.1.03	Tank	512 $\times$ 512	99.6273	33.4476
7.1.06	Truck and APCs	512 $\times$ 512	99.5945	33.5349
7.1.07	Tank	512 $\times$ 512	99.6185	33.4208
7.1.08	APC	512 $\times$ 512	99.6143	33.4797
7.1.09	Tank	512 $\times$ 512	99.6300	33.4495
7.1.10	Car and APCs	512 $\times$ 512	99.6044	33.4467

#### Information entropy analysis

The concept of information entropy quantifies the level of randomness or uncertainty inherent in an information source. A higher value of information entropy indicates a greater degree of uncertainty in the information source, making it more challenging for the proposed algorithm to predict or decipher the information. The information entropy *H*(*m*) of an information source *m* is computed using the following equation:

17 $$\begin{aligned} H(m)=\sum _{i=0}^{2^{n}-1} p(m_{i}) \log \frac{1}{p(m_{i})} \end{aligned}$$

where *M* is the total number of symbols $m(i)\in m$, $p(m_i)$ denotes the probability of a symbol.

Assuming that the source sends 256 symbols and that we can obtain the theoretical value $H(m)=8$ by using Eq. (17). The closer it is to 8, the less likely it is for an attacker to decode the cryptographic image. Table 3 shows the comparison of information entropy. From Table 3, we can see that the experimental results are close to 8, so the proposed algorithm has good information entropy properties.

**Table 3:** Image information entropy.

Image	Description	Size	Plaintext image	Proposed
4.1.01	Female (NTSC test image)	256 $\times$ 256	6.8981	7.9990
4.1.02	Couple (NTSC test image)	256 $\times$ 256	6.2945	7.9991
4.1.04	Female	256 $\times$ 256	7.4270	7.9991
4.1.05	House	256 $\times$ 256	7.0686	7.9991
4.1.06	Tree	256 $\times$ 256	7.5371	7.9991
4.1.07	Jelly beans	256 $\times$ 256	6.5835	7.9990
4.1.08	Jelly beans	256 $\times$ 256	6.8527	7.9991
4.2.01	Splash	512 $\times$ 512	7.2428	7.9998
4.2.03	Mandrill (a.k.a. Baboon)	512 $\times$ 512	7.7624	7.9998
4.2.05	Airplane (F-16)	512 $\times$ 512	6.6639	7.9998
4.2.06	Sailboat on lake	512 $\times$ 512	7.7622	7.9997
4.2.07	Peppers	512 $\times$ 512	7.6698	7.9998
5.1.09	Moon surface	256 $\times$ 256	6.7093	7.9990
5.1.10	Aerial	256 $\times$ 256	7.3118	7.9991
5.1.11	Airplane	256 $\times$ 256	6.4523	7.9991
5.1.12	Clock	256 $\times$ 256	6.7057	7.9989
5.1.13	Resolution chart	256 $\times$ 256	1.5483	7.9990
5.1.14	Chemical plant	256 $\times$ 256	7.3424	7.9992
5.2.09	Aerial	512 $\times$ 512	6.9940	7.9998
5.2.10	Stream and bridge	512 $\times$ 512	5.7056	7.9997
5.3.01	Male	1024 $\times$ 1024	7.5237	7.9996
5.3.02	Airport	1024 $\times$ 1024	6.8303	7.9999
7.1.01	Truck	512 $\times$ 512	6.0274	7.9998

### Image quality analysis

Peak signal-to-noise ratio (PSNR) and structural similarity (SSIM) are commonly used in the image processing field as a tool to weigh the quality of encryption. The mean square error (MSE) is part of PSNR and is defined as:

18 $$\begin{aligned} {\left\{ \begin{array}{ll} MSE=\frac{1}{H\times {W}}\sum _{H}^{i=1}\sum _{W}^{j=1}(X(i,j)-Y(i,j))^2\\ PSNR=10\times {\log _{10}(\frac{Q^2}{MSE})}\\ \end{array}\right. } \end{aligned}$$

where MSE denotes the mean square error of the plaintext image *X* and the ciphertext image *Y*, the height and width of the image are denoted by *H* and *W*, respectively, and *Q* denotes the pixel level of the image. SSIM is a measure of the similarity of two images and is defined as

19 $$\begin{aligned} SSIM(X,Y)=\frac{(2\mu _{X}\mu _{Y}+(0.01L)^2)(2\sigma _{XY}+(0.03L)^2)}{(\mu _{X}^2+\mu _{Y}^2+(0.01L)^2)(\sigma _{X}^2+\sigma _{Y}^2+(0.03L)^2)} \end{aligned}$$

where $\mu _{X},\mu _{Y}$ denotes the mean value of image *X* and *Y* respectively, $\sigma _{X}, \sigma _{Y}$ denotes the standard deviation of image *X* and *Y* respectively, and *L* denotes the dynamic range of the pixel values. The values of PSNR and SSIM are calculated using Eqs. (18) and (19) as shown in Table 4. The value of PSNR of an encrypted image should be around 30 dB, and the range of SSIM should be − 1 to 1. The closer the image is, the closer the absolute value of SSIM is to 1, so the value of SSIM should be above and below 0 after encryption.

**Table 4:** PSNR and SSIM values.

Images	Description	Size	PSNR	SSIM
4.1.01	Female (NTSC test image)	256 $\times$ 256	7.2943	0.0060
4.1.02	Female (NTSC test image)	256 $\times$ 256	6.2446	0.0038
4.1.04	Female	256 $\times$ 256	8.8212	0.0119
4.1.05	House	256 $\times$ 256	8.9060	0.0089
4.1.06	Tree	256 $\times$ 256	8.1694	0.0105
4.1.07	Jelly beans	256 $\times$ 256	8.5771	0.0108
4.1.08	Jelly beans	256 $\times$ 256	8.6537	0.0113
4.2.01	Splash	512 $\times$ 512	7.6306	0.0092
4.2.03	Mandrill (a.k.a. Baboon)	512 $\times$ 512	8.7607	0.0087
4.2.05	Airplane (F-16)	512 $\times$ 512	7.9782	0.0097
4.2.06	Sailboat on lake	512 $\times$ 512	8.0879	0.0082
4.2.07	Peppers	512 $\times$ 512	8.0743	0.0076
5.1.09	Moon surface	256 $\times$ 256	10.2123	0.0118
5.1.10	Aerial	256 $\times$ 256	9.2938	0.0112
5.1.11	Airplane	256 $\times$ 256	7.7650	0.0098
5.1.12	Clock	256 $\times$ 256	7.2930	0.0098
5.1.13	Resolution chart	256 $\times$ 256	4.9387	0.0070
5.1.14	Chemical plant	256 $\times$ 256	9.2117	0.0140
5.3.01	Male	1024 $\times$ 1024	8.0050	0.0085
5.3.02	Airport	1024 $\times$ 1024	8.7356	0.0092
7.1.01	Truck	512 $\times$ 512	9.9282	0.0106
7.1.02	Airplane	512 $\times$ 512	8.9670	0.0109
7.1.03	Tank	512 $\times$ 512	10.2004	0.0109
7.1.05	Truck and APCs	512 $\times$ 512	9.6001	0.0106
7.1.06	Truck and APCs	512 $\times$ 512	9.1209	0.0098
7.1.07	Tank	512 $\times$ 512	10.0524	0.0122
7.1.08	APC	512 $\times$ 512	10.3226	0.0105
7.1.09	Tank	512 $\times$ 512	9.8294	0.0111
7.1.10	Car and APCs	512 $\times$ 512	10.1723	0.0110

### Key space analysis

In cryptosystems, the easiest way to break the key is a brute force attack. Therefore, if the key space of the proposed algorithm is larger, the more difficult it is for the attacker to break the proposed algorithm by brute force attack. The size of the key space depends on the length of the security key, and it is one of the important factors to ensure the security of the cryptosystem. The chaotic system used in the image encryption algorithm designed in this paper, its key space can be expressed as $S\in \{\gamma ,MD5\}$, where $\gamma$ is the key parameter with an accuracy of $10^{-16}$ and *MD*5 is the hash value introduced to augment the key space, which produces a hash of 128 bits. The size of the key space of this encryption scheme is calculated to be about $10^{16}\times 2^{128}\approx 2^{181}$ and the key length reaches 181 bits. It can be seen from Table 5 that compared with other existing encryption schemes, the key space of this paper has obvious advantages.

**Table 5:** Key space comparison.

Ours	Ref.^79^	Ref.^80^	Ref.^81^	Ref.^82^	Ref.^83^
$2^{181}$	$2^{128}$	$2^{168}$	$2^{154}$	$2^{128}$	$2^{166}$

**Table 6:** Test results of key sensitivity.

Images	$10^{-12}$		$10^{-13}$		$10^{-13}$		$10^{-14}$	
	NPCR	UACI	NPCR	UACI	NPCR	UACI	NPCR	UACI
4.1.01R	99.6078	33.4426	99.6109	33.5864	99.6216	33.5878	99.6002	33.5384
4.1.01G	99.5789	33.4859	99.6170	33.3815	99.6475	33.4071	99.6155	33.2838
4.1.01B	99.5880	33.3818	99.5987	33.5874	99.5956	33.3428	99.5911	33.5640
4.1.04R	99.6429	33.4931	99.5712	33.5379	99.6078	33.5110	99.6307	33.5426
4.1.04G	99.6338	33.6469	99.6140	33.4711	99.6155	33.3754	99.6124	33.5905
4.1.04B	99.5422	33.4235	99.6155	33.4438	99.6307	33.4151	99.5728	33.6807
4.1.05R	99.5758	33.4490	99.5972	33.3425	99.6277	33.4155	99.5865	33.4755
4.1.05G	99.6078	33.4262	99.5895	33.4607	99.5972	33.4287	99.6262	33.5700
4.1.05B	99.6246	33.3048	99.6048	33.3665	99.6414	33.5043	99.6033	33.5037
4.1.06R	99.6277	33.3131	99.6399	33.4018	99.5850	33.5176	99.5972	33.5695
4.1.06G	99.5895	33.4885	99.5941	33.3695	99.6002	33.5800	99.6078	33.4090
4.1.06B	99.5621	33.5960	99.5667	33.4374	99.6201	33.3366	99.6567	33.4157
4.1.07R	99.6231	33.6294	99.6048	33.3397	99.5850	33.4698	99.6277	33.5210
4.1.07G	99.5834	33.5737	99.5926	33.4378	99.5972	33.4821	99.6368	33.5756
4.1.07B	99.6277	33.4999	99.5728	33.6140	99.6414	33.5406	99.6002	33.5816
4.2.01R	99.6124	33.4470	99.6243	33.5259	99.6101	33.5210	99.6212	33.3466
4.2.01G	99.6014	33.4142	99.6063	33.5191	99.6231	33.4901	99.6082	33.5039
4.2.01B	99.6189	33.5039	99.6075	33.4554	99.6288	33.4667	99.6094	33.4710
4.2.03R	99.6136	33.4783	99.6227	33.4670	99.6071	33.4185	99.6101	33.4253
4.2.03G	99.6113	33.4052	99.6113	33.3953	99.5869	33.4642	99.5911	33.4488
4.2.03B	99.6159	33.4468	99.6098	33.5104	99.6006	33.4174	99.5987	33.4489
4.2.05R	99.6281	33.4984	99.6334	33.5256	99.6037	33.3911	99.6113	33.3996
4.2.05G	99.5987	33.4142	99.6170	33.4377	99.6189	33.4837	99.6086	33.4805
4.2.05B	99.5892	33.4265	99.6197	33.4010	99.6002	33.3928	99.6040	33.3521
4.2.06R	99.6254	33.4345	99.6143	33.4290	99.6162	33.4800	99.6201	33.4109
4.2.06G	99.5960	33.4937	99.6273	33.5904	99.6109	33.5504	99.6086	33.4114
4.2.06B	99.6113	33.4053	99.6155	33.3712	99.6056	33.4204	99.6029	33.4491
4.2.07R	99.6170	33.5162	99.6269	33.4951	99.6086	33.5300	99.6078	33.4584
4.2.07G	99.6155	33.4870	99.6117	33.4947	99.6075	33.4331	99.6090	33.4854
4.2.07B	99.6204	33.4846	99.6063	33.4295	99.6078	33.4619	99.6223	33.4850
4.1.07R	99.6231	33.6294	99.6048	33.3397	99.5850	33.4698	99.6277	33.5210
4.1.07G	99.5834	33.5737	99.5926	33.4378	99.5972	33.4821	99.6368	33.5756
4.1.07B	99.6277	33.4999	99.5728	33.6140	99.6414	33.5406	99.6002	33.5816
4.2.01R	99.6124	33.4470	99.6243	33.5259	99.6101	33.5210	99.6212	33.3466
4.2.01G	99.6014	33.4142	99.6063	33.5191	99.6231	33.4901	99.6082	33.5039
4.2.01B	99.6189	33.5039	99.6075	33.4554	99.6288	33.4667	99.6094	33.4710

### Sensitivity analysis

In this section, the sensitivity performance of the algorithm is analyzed in terms of the sensitivity of the key and plaintext, respectively. A secure algorithm should be highly sensitive, which means that if there is a slight change in the key or plain image information during encryption or decryption, the correct result cannot be obtained.

#### Key sensitivity analysis

**Figure 9:**
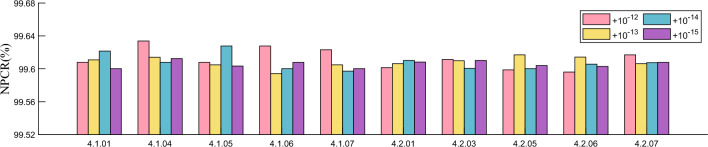
Test values of NPCR after different keys are perturbed.

**Figure 10:**
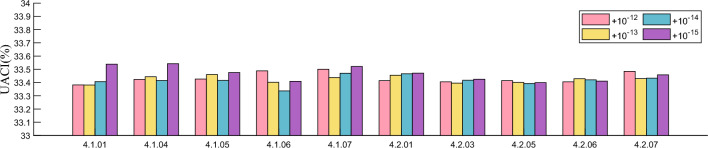
Test values of UACI after different keys are perturbed.

It is a property that good cryptosystems should have that the key used does not yield the correct ciphertext even if there is a small difference. In this section, we compare the differences between the ciphertexts obtained by encrypting with the correct key and the slightly changed keys ($+10^{-12}$, $+10^{-13}$, $+10^{-14}$, $+10^{-15}$). The difference between them is derived by calculating NPCR and UACI, where NPCR and UACI are calculated as shown in Eq. (15). The results are shown in Table 6 and Figs. 9 and 10, where we can find that the average values of NPCR and UACI are 99.6108% and 33.4707% respectively when the perturbation is added to the key. This indicates that the difference between the two cipher images is very large. Hence the proposed algorithm in this paper has good encryption results.

#### Analysis of plaintext sensitivity

**Figure 11:**
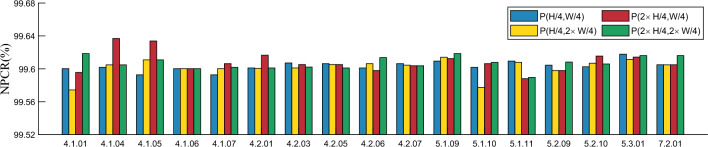
Test values of NPCR after different keys are perturbed.

**Figure 12:**
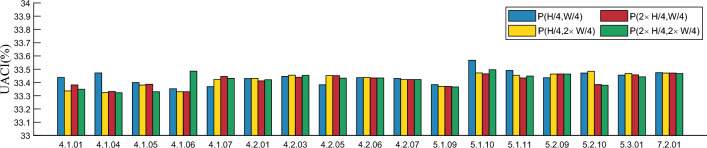
Test values of UACI after different keys are perturbed.

In good encryption algorithms, even a small change in the plaintext image can make the encrypted ciphertext image look very different from the ciphertext encrypted from the unchanged plaintext image. If the proposed algorithm does not have this ability, it is very easy for an attacker to break the algorithm by analyzing the correlation between the plaintext image and the ciphertext image. Therefore, the plaintext image sensitivity of an algorithm is the key to its resistance to plaintext image attacks. In this section, we analyze the sensitivity of the proposed algorithm to plaintext images by adding 1 to the pixel values of plain images at (H/4, W/4), (H/4, W $\times$ 2/4), (H $\times$ 2/4, W/4), and (H $\times$ 2 /4, W $\times$ 2/4) to compute the NPCR and UACI. the results are shown in Table 7 and Figs. 11 and 12. Table 7 demonstrates that when pixel values change by 1 at specific locations, the average NPCR between the corresponding ciphertext images and the original ciphertext images approximate the ideal value of 99.6%. Additionally, the average UACI value closely resembling the ideal value of 33.4%. These results signify a prominent alteration in the cryptographic image and render the algorithm robust against plaintext attacks.

**Table 7:** Test results of plaintext sensitivity.

Image	(*H*/4, *W*/4)		$(H/4,W\times {2}/4)$		$(H\times {2}/4,W/4)$		$(H\times {2}/4,W\times {2}/4)$	
	NPCR	UACI	NPCR	UACI	NPCR	UACI	NPCR	UACI
4.1.01R	99.6002	33.5242	99.5712	33.4215	99.6109	33.5043	99.5926	33.4215
4.1.01G	99.5590	33.4577	99.5697	33.3357	99.5911	33.3809	99.6399	33.3485
4.1.01B	99.5850	33.4375	99.5743	33.6102	99.5956	33.5468	99.6185	33.5854
4.1.04R	99.6353	33.6232	99.6048	33.5286	99.5865	33.5072	99.6048	33.4962
4.1.04G	99.6170	33.5945	99.5956	33.3239	99.6368	33.3324	99.6429	33.3227
4.1.04B	99.6017	33.4719	99.5911	33.4921	99.6368	33.4966	99.6216	33.5071
4.1.05R	99.5926	33.3990	99.6170	33.3803	99.6399	33.3868	99.6124	33.3998
4.1.05G	99.6338	33.5469	99.6109	33.5500	99.6338	33.5533	99.6109	33.5500
4.1.05B	99.5743	33.4945	99.6674	33.5171	99.5697	33.4928	99.6674	33.5171
4.1.06R	99.5544	33.3527	99.6201	33.3301	99.6201	33.3299	99.5880	33.3297
4.1.06G	99.6002	33.4921	99.6002	33.5148	99.6002	33.5148	99.6002	33.5148
4.1.06B	99.6475	33.4935	99.6033	33.4906	99.6033	33.4906	99.6033	33.4906
4.1.07R	99.6216	33.4898	99.6002	33.4670	99.5865	33.4889	99.6017	33.4851
4.1.07G	99.6216	33.6001	99.6109	33.4222	99.6063	33.4457	99.5987	33.4683
4.1.07B	99.5926	33.3688	99.6201	33.6785	99.6185	33.6446	99.6231	33.6610
4.2.01R	99.6021	33.4393	99.5762	33.4374	99.5956	33.4281	99.6235	33.4309
4.2.01G	99.5991	33.4729	99.6201	33.4798	99.6166	33.4659	99.5865	33.4867
4.2.01B	99.6010	33.4293	99.6006	33.4313	99.6170	33.4122	99.6010	33.4388
4.2.03R	99.6319	33.4601	99.5922	33.4610	99.6113	33.4397	99.5888	33.4207
4.2.03G	99.6071	33.4465	99.6010	33.4552	99.6174	33.5002	99.6063	33.4903
4.2.03B	99.6166	33.4925	99.5983	33.4665	99.6052	33.4801	99.6021	33.4538
4.2.05R	99.6063	33.3824	99.5930	33.4763	99.5930	33.4767	99.6155	33.4737
4.2.05G	99.6136	33.4661	99.6052	33.4798	99.6052	33.4798	99.6185	33.4588
4.2.05B	99.6189	33.4509	99.6056	33.4516	99.6056	33.4516	99.6010	33.4323
4.2.06R	99.6010	33.4915	99.6143	33.4867	99.6128	33.4869	99.6185	33.4848
4.2.06G	99.6193	33.4365	99.6063	33.5326	99.6136	33.5440	99.6136	33.5440
4.2.06B	99.5991	33.4361	99.6140	33.4379	99.5979	33.4338	99.5979	33.4338
4.2.07R	99.6136	33.4305	99.6048	33.4433	99.6048	33.4436	99.6044	33.4431
4.2.07G	99.6281	33.4893	99.6044	33.4215	99.6037	33.4221	99.6037	33.4221
4.2.07B	99.6063	33.5253	99.6296	33.5224	99.6155	33.4440	99.6155	33.4440
5.1.09	99.6094	33.3835	99.6140	33.3703	99.6124	33.3702	99.6185	33.3663
5.1.10	99.6017	33.5678	99.5773	33.4721	99.6063	33.4650	99.6078	33.4956
5.1.11	99.6094	33.4905	99.6078	33.4540	99.5880	33.4337	99.5895	33.4482
5.2.09	99.6044	33.4352	99.5979	33.4637	99.5979	33.4639	99.6082	33.4633
5.2.10	99.6025	33.4710	99.6067	33.4840	99.6155	33.3845	99.6059	33.3791
5.3.01	99.6178	33.4549	99.6113	33.4673	99.6143	33.4572	99.6161	33.4423
7.2.01	99.6049	33.4738	99.6048	33.4707	99.6049	33.4707	99.6161	33.4675

### Execution time analysis

This research aimed to assess the performance of the proposed encryption algorithm concerning image encryption across various sizes. Four sets of images were chosen, sized at $64 \times 64$, $128 \times 128$, $256 \times 256$, and $512 \times 512$ pixels, respectively. Table 8 illustrates the average runtime for encryption across different image sizes. The intent was to highlight the encryption performance of the algorithm across varying image dimensions using intuitive visualizations.

**Table 8:** The encryption times of different algorithms (unit: s).

Size	Ours	Ref.^84^	Ref.^85^	Ref.^86^
$64 \times 64$	0.010171	0.011306	–	–
$128 \times 128$	0.033372	0.039643	0.0687	–
$256 \times 256$	0.156314	0.167471	0.2637	0.382
$512 \times 512$	0.638506	0.732927	1.1003	1.489

### Robustness analysis

Robustness measures whether the algorithm can effectively withstand interferences, safeguarding the image content from damage or leakage. In practical applications, images may encounter diverse interferences, making a thorough analysis and evaluation of encryption algorithms against these interferences significantly crucial. In this section, we have selected salt and pepper noise and clipping attack as the subjects of study to explore their impact on image encryption algorithms.

#### Salt and pepper noise analysis

**Figure 13:**
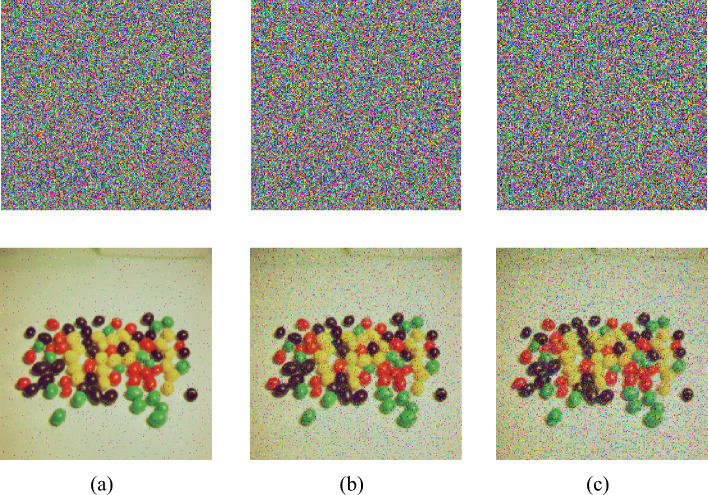
The ciphertext and decryption image after adding salt and pepper noise: (**a**) 0.01, (**b**) 0.05, (**c**) 0.1.

Noise attacks represent an alternative method for manipulating images, involving the application of statistical techniques to alter specific points within the image. These alterations are often subtle and challenging to detect. Therefore, an effective image encryption algorithm should demonstrate robust resilience against noise attacks. In this study, salt and pepper noise is utilized as the attacking method, as depicted in Fig. 13. The research findings indicate that the encryption algorithm exhibits substantial resistance against noise attacks.

#### Clipping attack analysis

**Figure 14:**
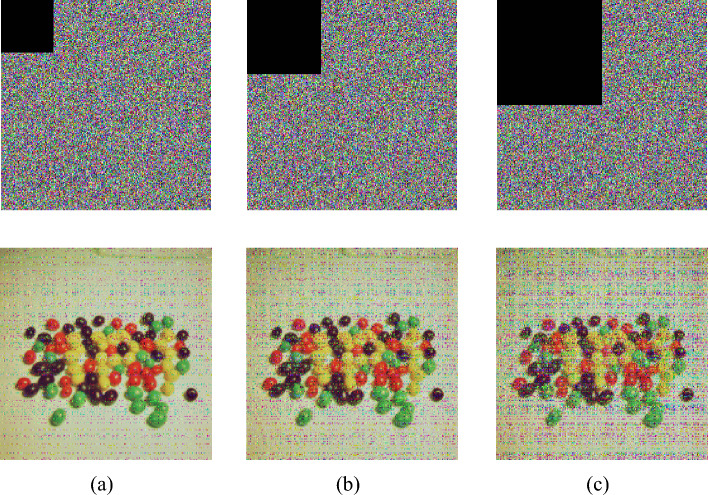
The ciphertext and decryption image after adding clipping noise: (**a**) 1/16, (**b**) 1/8, (**c**) 1/4.

During communication, if signal interception occurs, the transmitted ciphertext might be tampered with. To prevent such scenarios, ciphertexts should possess strong resistance against clipping attack. We conducted clipping attack at rates of 1/16, 1/8, and 1/4 at different positions, and used the resulting clipped ciphertexts for decryption. As shown in Fig. 14, the encryption algorithm demonstrates robust resilience against clipping attacks. Even when the clipping ratio reaches 1/4, the primary content of the image remains visible.

## Conclusion

This paper proposes a bit-level image privacy protection scheme using Zigzag and chain-diffusion, it can enhance the ability of image privacy protection scheme to resist password attacks. The scheme adopts the strategy of encryption by weight for each bit layer and designs a chain diffusion method of Zigzag interleaving scrambling with hash value plaintext association. At the same time, non-sequential encryption is adopted to achieve efficient and secure encryption effect. To improve security performance, we introduce a hash-generated chaotic sequence to encrypt each bit layer. The generation of chaotic sequences depends on the hash value of the previous bit, which ensures that the encryption process of each bit layer is independent. Finally, we use a non-sequential encryption technique to non-linearly rearrange the bit ciphertext image, which further enhances the encryption effect. Each encryption module adopts the forward plaintext feedback encryption mechanism, which effectively enhances the avalanche effect of the cipher. The results show that the scheme has robustness and significant diffusion properties and can successfully resist various common cipher attacks. The scheme proposed in this paper combines the features of digital images layered by bit with different visual weights and both considers the security and efficiency of image privacy protection, and thus is a preferred technical solution. Especially in the context of big data era, the technical scheme has potential practical application value.

## Data Availability

The datasets used and analysed during the current study available from the corresponding author on reasonable request. All data generated or analysed during this study are included in this published article.
